# Soils and Sanitation

**Published:** 1913-01-04

**Authors:** 


### HOSPITAL ARCHITECTURE AND CONSTRUCTION.

### Soils and Sanitation.

The bases of walls and piers directly supported or kept in equilibrium by the earth are called foundations, and their failures are caused by inequalities of the load distribution or of the earth resistance. It is almost impossible to guarantee against minor movements in some soils, as nearly all are compressible; but care must and can be taken to ensure that the movement will be uniform and of no serious consequence, such as would cause rupture of the structure. This is most essential where the building has concentrated loads at intervals only on the ground, as distinct from the type where all the walls rest on one supporting concrete platform of equal thickness and equivalent width. In a foundation upon running sand or peaty soil, the weight of the building causes the ground to be squeezed out from under the foundation; unless it is confined in some manner, such as by sheet piling. Sheet piling consists of elm timbers, say, 3 inches thick and 9 to 11 inches in width, with square solid guide posts, say, 9 inches square at 6 to 10 feet intervals. The piles may be any reasonable length and each has an iron-pointed shoe and strap-iron head band; they are all joggled one into another, or in some other manner connected together, so that where the piling operations are completed the enclosed area has a strong belt of solid timber which prevents the lateral escape of the earth. This method of consolidating the earth (or by substituting reinforced concrete piling for wood piling) may also be adopted on a hillside where the tendency of the earth is to slide in bulk, owing to the pressure put upon it: in many cases the erection of a retaining wall will answer the purpose better, as it does not shake and disturb the soil.

When piles are driven to confine an area it is usual to drive others in the enclosure at intervals to consolidate the ground and in some degree transmit the pressure to the solid and more reliable strata beneath; thus Rankine, an acknowledged classic on the subject, gives, from practical examples, 1,000 lb. per square inch of area of head of pile as a safe load to work upon, provided of course that the pile reaches a secure bearing and is vertically driven. Where the pile does not reach a firm bottom, but bears only by friction on its sides, one-fifth of the former figure should be worked to as a safe load.

Buildings erected on damp soils, such as a sand overlying clay, have their stability impaired if water be drained away from about the foundations after the building has taken its bearing. This is evident, for if the foundation soil has water taken from its bulk the remainder must be compressible into less compass. Provision must, therefore, be made either for keeping the soil free from water or to ensure that the amount of water will not be materially lessened. The latter is unfortunately not very easy to provide for, and probably that is why so many ruptures in comparatively light buildings occurred during and immediately following the summer of 1911. Owing to the hot and dry weather the bulk of water in water-bearing strata was reduced very materially, and naturally buildings went down with the surfaces on which the foundations rested.

Many otherwise reliable soils are much deteriorated by being exposed to the atmosphere and to rainfall during the erection of a building, and it is necessary in such cases to put the foundations at a depth from the surface so that the underlying soil will not be subjected to percolation and subsequent freezing of surface water. This depth rarely exceeds 4 feet, and 3 feet is still more common.

The construction of a foundation varies with the bearing resistance of a soil. Rock, sand, gravel, clay, and chalk will all vary in the method of treatment to sustain even a moderate-sized building.

Rock generally is a good and secure foundation, which when levelled may be built upon without any concrete base to distribute the thrust of the walls; but it is liable to faults or fissures, which must be hewn out and filled in with concrete. Rock foundations are generally expensive owing to the extra labour involved. Sand, when it can be kept dry and confined laterally, forms a good foundation, but if subject to running water is very much weakened.

Gravel is one of the best soils to build upon if lateral movement is not likely to occur; it is free from atmospheric action and is practically incompressible.

Clay is sensitive to atmospheric influence, but otherwise is good to build upon provided the foundation is carried deep enough to be beyond the action of the atmosphere. Chalk foundations are very variable; if dry or well drained they are very hard, but if subject to too much wet they are unreliable. Precaution must, therefore, be taken to prevent the entrance of too much moisture, and generally a concrete base is necessary to distribute the pressure over a comparatively large area.

The sanitation of soils is of the utmost importance to those who live in proximity to them. A soil may be detrimental to health by its dampness or by the growth or putrescence of vegetable matter. All other conditions being equal, low-lying districts are not so healthy as those on high ground or hill slopes where good natural drainage is obtainable.

Dryness or porosity of soil is an important factor; where this is not provided by Nature it may be conduced to artificially. Dampness of soil is detrimental to health, and tends to cause defects in buildings by expansion and contraction consequent upon the absorption and evaporation of moisture. It is possible to keep a site comparatively free from water by laying a system of deep and narrow trenches 10 to 30 feet apart, the bottoms of which are filled with gravel, broken stones, or other material so that water may percolate easily through till it is conducted beyond the area occupied by inhabited buildings.

A layer of concrete is required by the building bye-laws of most districts over the whole area of buildings erected, to prevent organic growth and the emanation of ground gases. In hospital planning this requirement has been further elaborated by laying a concrete bed over the whole area and by the construction of the ground floor a few feet higher, so that a thorough and efficient blow through of fresh air is obtained.

Building bye-laws also require that walls shall have an efficient horizontal damp-course of slates, asphalte, or other approved material. Damp may further be guarded against by the erection of walls with a hollow space in their centres, and it should not be forgotten that provision should be made for draining the hollow space where water might accumulate. Such walls are particularly desirable where an equable temperature is desired, as in the case of a hospital ward; and especially so on the wet aspects, such as south-west; but they do not seem to be in strong favour with hospital architects for some unknown reason.

